# The Diagnostic Value of TTF-1, P63, HMWK, CK7, and CD56 Immunostaining in the Classification of Lung Carcinoma

**Published:** 2017-07-01

**Authors:** Amir Hossein Jafarian, Masoumeh Gharib, Nema Mohammadian Roshan, Samaneh Sherafatnia, Abbas Ali Omidi, Sahar Bagheri

**Affiliations:** 1 *Dept. of Pathology, Faculty of Medicine, Mashhad University of Medical Sciences, Iran*

**Keywords:** Immunohistochemistry, TTF1, P63, HMWK, CK7, CD56, lung carcinoma

## Abstract

**Background & objective:**

The histologic distinction of small cell from non-small cell lung carcinoma and correct identification of all subtype**s **of lung carcinoma are very important in treatment management. The main method for histologic classification of lung tumors is based on morphology. However, in small bronchoscopic biopsies in particular, distinction is very difficult upon morphology alone. The current study aimed at evaluating the utility of a panel of antibodies, consisting of thyroid transcription factor (TTF-1), P63, high molecular weight keratin [HMWK (34βE12)], cytokeratin (CK7), and cluster of differentiation (CD56) for accurate distinction of bronchogenic carcinomas.

**Methods:**

Bronchoscopic biopsies of 60 lung carcinoma cases including 20 small cell carcinomas, 20 adenocarcinomas, and 20 squamous cell carcinomas (SCCs) with typical morphologic features were selected. All these cases were immunohistochemically stained for TTF-1, P63, HMWK (34βE12), CK7, and CD56. All immunostained slides were scored as either positive or negative.

**Results:**

The mean age of the patients was 60 years; ranged from 35 to 81. Sixteen patients were female and 44 were male. All adenocarcinomas were positive for CK7 and most of them (18/20; 90%) were positive for TTF-1. Most of small cell lung carcinomas were positive for TTF-1 (17/20; 85%), and CD56 (18/20; 90%). All squamous cell carcinomas (SCCs) were negative for TTF-1, but most of them were positive for HMWK (34βE12) and P63.

**Conclusion:**

The obtained data showed that TTF-1, P63, CK7, CD56 and/or 34βE12 represent a useful panel of antibodies to identify lung carcinoma subtypes in small bronchoscopic biopsies.

## Introduction

Worldwide, 1.8 million patients were diagnosed with lung cancer in 2012 that caused an estimated 1.6 million deaths (1). In the United States, there are approximately 225000 new cases of lung cancer and over 160000 deaths annually (2). Around 1953, lung cancer became the most common cause of cancer deaths in males, and in 1985, it became the leading cause of cancer deaths in females. However, due to decreased smoking habits, there is a decline in lung cancer deaths in both genders (3). The 2015 World Health Organization (WHO) classification that should be the foundation for lung cancer recognizes four major histologic cell types(4): Adenocarcinoma (including bronchioalveolar carcinoma), squamous cell carcinoma, large cell carcinoma, and small cell carcinoma. Compared to previous classification systems, to a greater extent it relies on immunohistochemistry to subtype lung cancers and provides standardized criteria and terminology to diagnose small biopsies and cytology, which are the main sampling methods in patients with high-stage cancers. The 2015 WHO classification also has guidelines to perform molecular studies that are crucial in the targeted therapies (4).

Morphological assessment of hematoxylin-eosin histological sections is still the main method to classify lung cancer, but this approach may be difficult or even non-practical in cytological preparations or small biopsies. Moreover, in the era of the targeted therapy, the mere differentiation of small-cell carcinoma from non-small cell carcinoma (NSCLC) is too simplistic. In poorly-differentiated NSCLCs, more accurate characterization of NSCLC is very hard. However, the incorporation of an immunohistochemical panel including markers of squamous such as high-molecular-weight cytokeratins HMWK [34βE12] and P63 and glandular cell differentiation markers such as TTF-1, and cytokeratin 7 differentiation seems promising.

The current study aimed at evaluating the utility of a panel of antibodies including TTF-1, P63, HMWK (34βE12), CK7, and cluster of differentiation (CD56) for accurate distinction of bronchogenic carcinomas.

## Material and Methods

The present study was performed on formalin-fixed, paraffin-embedded (FFPE) tissue samples of 128 patients diagnosed with pulmonary adenocarcinoma (ADC), small cell carcinoma (SmCC), and squamous cell carcinoma (SCC) diagnosed and treated from 2001 to 2009 in Ghaem Hospital, a major referral center in Mashhad, Iran. Hematoxylin and eosin (H&E)-stained slides were reviewed by three pathologists, using three-headed microscope (Nikon light microscope, Japan) to confirm the diagnosis (according to the 2015 WHO classification system). The inclusion criteria were the presence of morphological characteristics for these three subtypes and enough FFPE tumoral tissue for immunohistochemical staining. Specimens with extensive necrosis, hemorrhage, and crush artifact were excluded from the study. After applying the inclusion and exclusion criteria, 60 appropriate FFPE blocks were selected, including 20 ADC, 20 SmCC, and 20 SCC specimens. Immunohistochemical staining was performed on 5-μm tissue sections for five markers (TTF-1, P63, HMWK (34βE12), CK7, and CD56). All antibodies were ready for use except TTF-1, which was diluted to 1:25. All antibodies were made by DAKO Company except CD56 (Novocastra, Newcastle, UK). Bronchial epithelium was used as positive internal control for P63, HMWK (34βE12), and CK7, and pulmonary alveoli for TTF-1. Moreover, SmCC was used as positive control for TTF-1 and CD56. Negative control was assessed by not adding the primary antibody. All of the immunostained slides were scanned at 400X magnification to investigate tumor cells overall distribution. Samples were considered positive if at least 10% of tumor cells showed nuclear staining for TTF-1 and P63, cytoplasmic staining for HMWK (34βE12) and CK7, and cytoplasmic and membranous staining for CD56 (cutoff point of 10%, based on the study by Rossietal.) (11).

## Ethical Approval

*The current study protocol was approved by the *Institutional Review Board *(IRB) and Ethics Committee of *Mashhad University of Medical Sciences (MUMS)*.*

## Results

A total of 60 FFPE tissue blocks with the definite diagnosis of bronchogenic carcinoma (BC) were analyzed. The project yielded the following results:

BC appears to be more common in males (male to female ratio: 2.75: 1). The mean age of the patients was 60 years; ranged from 35 to 81.The mean age of males was greater than that of females, although the difference was statistically insignificant (P-value > 0.05). Moreover, the mean age of diagnosis in the three BC subtypes was insignificant (P-value>0.05). There was a statistically significant positive correlation between the gender and BC subtypes (P-value = 0.003). According to the obtained results, 9 out of 16 female subjects (56.2%) and 11 out of 44 male subjects (25%) had adenocarcinoma. All of the SCC specimens belonged to male subjects. Staining distribution of all immunohistochemical markers was strongly associated with BC subtypes (P-value <0.0001).

**Figure1:**
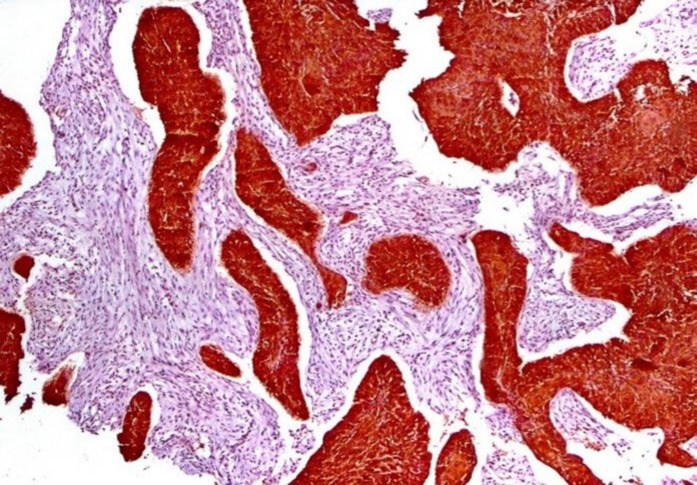
Positive 34βE12 Immunostaining in Squamous Cell Carcinoma

Immunohistochemical study revealed the following results: 

34βE12, with 100% sensitivity, 80% specificity, 100% positive predictive value (PPV), and 71.4% negative predictive value (NPV) was a good marker to differentiate SCC from ADC and SmCC (P-value<0.0001) (Figure 1). 

**Figure 2:**
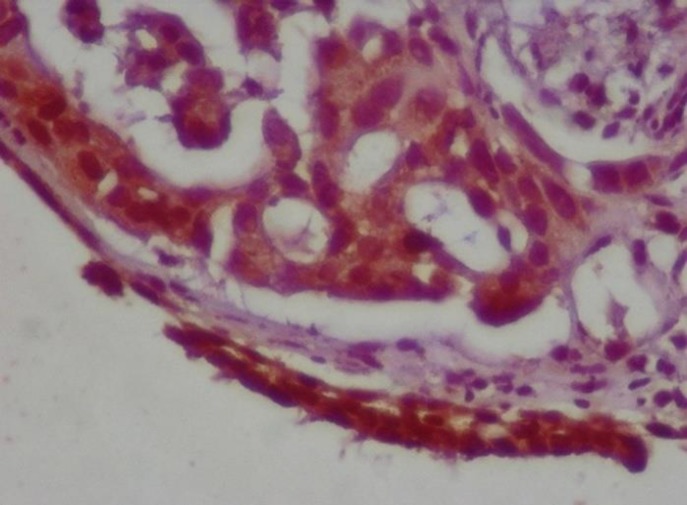
Positive CK7 Immunostaining in Adenocarcinoma.

**Figure 3:**
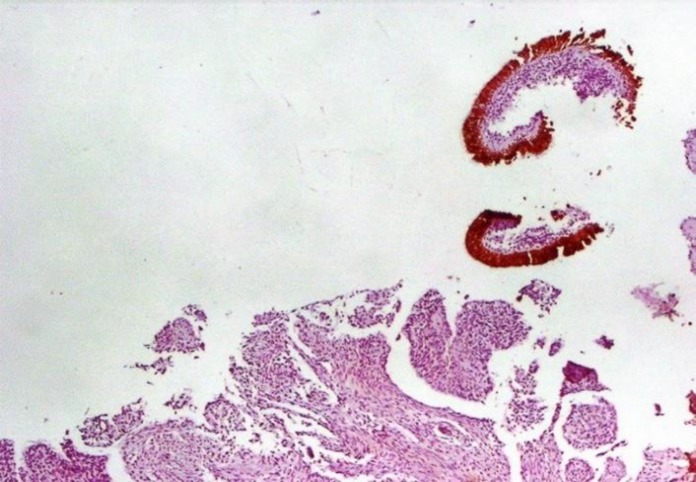
Negative CK7 Immunostaining in Squamous Cell Carcinoma and Positive Internal Control

**Figure 4:**
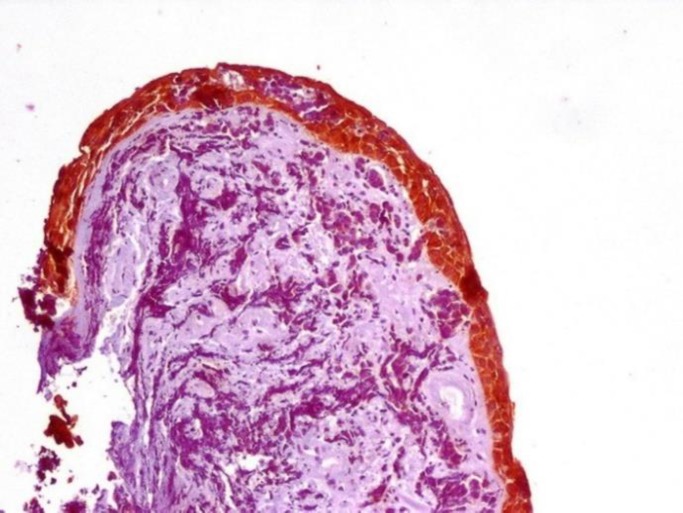
Negative CK7 Immunostaining in Small Cell Carcinoma and Positive Internal Control

CK7, with 100% sensitivity and 92.5% specificity, was a very good marker to differentiate ADC from the other two BC subtypes (figures 2 to 4).

**Figure 5:**
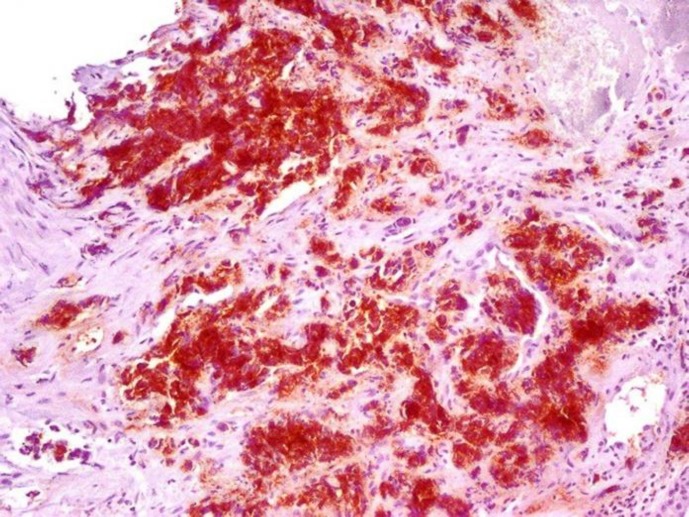
Positive CD56 Immunostaining in Small Cell Carcinoma.

CK7 negativity ruled out the diagnosis of ADC. CD56, with 90% sensitivity, 100% specificity, 100% PPV, and 95.2% NPV, was a good marker to differentiate SmCC from SCC and ADC (P-value< 0.0001) (Figure 5).

**Figure 6:**
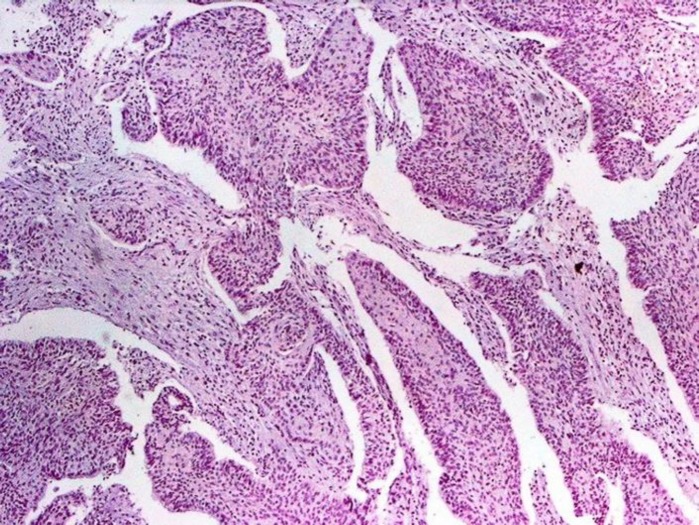
Negative TTF-1 Immunostaining in Squamous Cell Carcinoma

Positive TTF-1, with 85% sensitivity, 100% specificity, 100% PPV, and 87% NPV, was beneficial for SmCC diagnosis, whereas its sensitivity and PPV for SCC diagnosis were zero (Figure 6).

**Figure 7:**
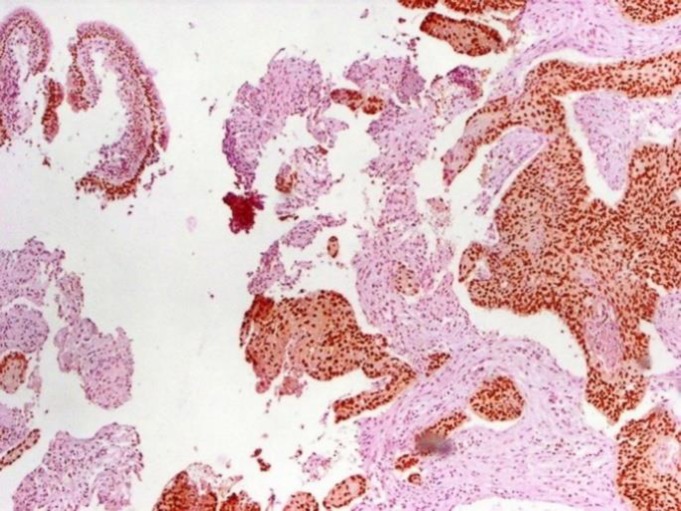
Positive P63 Immunostaining in Squamous Cell Carcinoma

**Figure 8:**
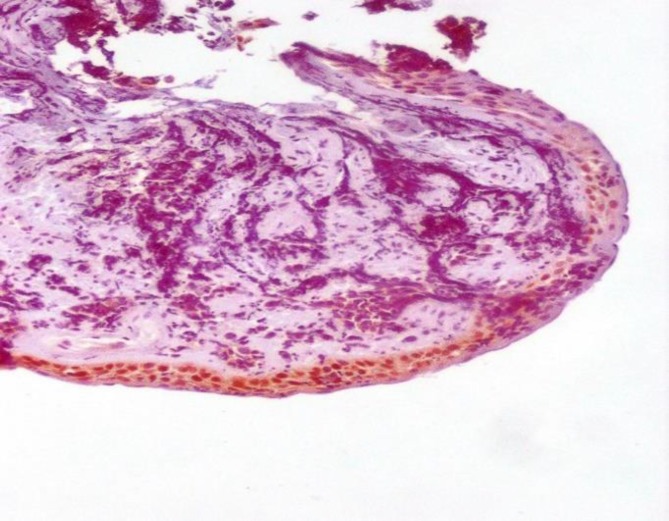
Negative P63 Immunostaining in Small Cell Carcinoma

P63, with 85% sensitivity, 98% specificity, 94.4% PVV, and 86.4% NPV, differentiated SCC from SmCC (figures 7 and 8).

The 34βE12, with 100% sensitivity and specificity, differentiated SCC from SmCC. CD56, with 90% sensitivity, 100% specificity, 100% PPV, and 90.9% NPV, differentiated SmCC from SCC. CK7, with 100% sensitivity and specificity, differentiated ADC from SmCC. TTF-1 staining had 90% sensitivity for ADC and 95% sensitivity for SmCC, but did not have good specificity to differentiate the two types. Therefore, it was not a good marker for differential diagnosis of ADC and SmCC.

## Discussion

Since the prognosis and treatment of BC are related to its subtype, differentiating SmCC from non-SmCC and accurate diagnosis of each subtype is of utmost importance. Generally, SmCC is treated by radiotherapy and non-SmCC by surgery. Only 1/3 of non-SmCC cases are cured by surgery and 2/3 (unresectable cases) need chemotherapy. Moreover, the non-SmCC subtype (SCC, LCC, and ADC) affects the type of chemo/radiotherapy. It is known that the treatment of SCC is different and its prognosis is better. Among non-SmCC subtypes, ADC has a better response to epidermal growth factor receptor inhibitor. Nowadays, cytology and bronchoscopic biopsies are used to diagnose and classify BC. In the presence of adequate tissue and characteristic features, such as squamous or glandular differentiation, the diagnosis is feasible. However, in cases with poor differentiation and especially in small bronchoscopic biopsies, the diagnosis becomes difficult. These small biopsies may not have enough tumoral cells for the morphological classification of bronchial tumors, which makes the diagnosis difficult. Differentiation of SmCC and poorly-differentiated non-SmCC can become even more difficult in the presence of necrosis, crush artifact, inadequate tissue fixation, and small cells with little cytoplasm. Therefore, it is important to use a reliable, sensitive, and specific procedure to classify BC subtypes. 

The current study used five markers: TTF-1, P63, CK7, 34βE12, and CD56 to differentiate SCC, ADC, and SmCC. 

Previous studies revealed TTF-1 positivity in 40% to 100% of ADC specimens and 61% to 100% of SmCC specimens. This marker is almost always negative in SCC specimens. Karji et al., showed the lowest positivity for TTF-1 in ADC (40%). In their opinion, that result was due to small bronchoscopic biopsies and the larger specimens may yield more positive results(5). In the current study TTF-1 was positive in 18/20 of ADCs (90%) and 17/20 of SmCCs (85%), and similar to the previous studies, it was negative in 20/20 of SCCs. TTF-1 was a sensitive marker to diagnose ADC and SmCC (90% and 85% sensitivity, respectively), but was not specific. It was not a useful marker to differentiate between ADC and SmCC. To distinguish ADC from SmCC, morphological feature scan be helpful, but if it did not, other markers such as CK7 and CD56 can be used.

In other studies, P63, a P53 homolog, was considered as a good marker to diagnose SCC, especially in poorly-differentiated SCC. It was mostly expressed in the margins of squamous nests in poorly-differentiated SCC, and similar toTTF-1 had a nuclear staining pattern. Therefore, it was more reliable than neuroendocrine markers such as synaptophysin and chromogranin, which had cytoplasmic expression. P63 can be expressed a little in other BCs. In Au N study, P63 was positive in 30% of ADC and 76.9% of SmCC cases (6), but it was not positive in other studies including the current one. The reason for P63 expression in the study by Au N may be due to the technic and dilution of antibodies. Long incubation time in low temperature (overnight at 4°**C**) against the conventional method (one hour at room temperature) can lead to increased sensitivity for P63. In the study by Wang, P63 was positive in 65% of ADC cases. However, it was focal and weak in comparison with the strong and diffuse staining of poorly-differentiated SCCs(7). In other studies, none of the ADCs and SmCCs expressed P63, whereas all SCCs expressed P63. In the study by karji, P63 became positive in 32/39 specimens of SCC(5). In the current study, P63 was negative in all ADC specimens, but became positive in 1/20 of SmCCs (5%), and 17/20 of SCCs (85%). P63, with 85% sensitivity and 97.5% specificity, can distinguish SCC from other subtypes. It can distinguish SCC from ADC with 85% sensitivity, 95% specificity, 94.4% PPV, and 86.4% NPV, and distinguish SCC from SmCC with 85% sensitivity, 100% specificity, 100% PPV, and 87% NPV. Therefore, it was a good marker to distinguish SCC from NSCC. CK7 was a glandular differentiation marker and was expressed in 60% to100% of ADCs in the previous studies. It was not expected to be expressed in SCCs and SmCCs. However, in the studies by Marson, Johansson, and Lyda, CK7 was expressed in approximately one fourth of SCCs (8-10). In the present study, CK7 could diagnose ADCs with 100% sensitivity. It was positive in none of the SmCCs, but positive in 3/20 of SCCs (15%).

The 34βE12 is a cytoplasmic marker expressed in pulmonary basal cells, bronchial epithelium, and SCCs. In the study by Rossi, the tumors that expressed only 34βE12 (as opposed to CD56, CK7, and TTF-1) were considered SCC(11). In the study by Lyda, 34βE12 was positive in 1/31 of SmCCs (3.7%), 41/42 of SCCs (97.6%) and 14/33 of ADCs (42%)(10). The 34βE12 is not usually expressed in SmCCs. However, in the studies by Sturm and Viberti, it was positive in 12.5% and 6.9% of SmCCs, respectively(12, 13). In the current study, 34βE12 was positive in 100% of SCCs and negative in 100% of SmCCs. Therefore, 34βE12 with the sensitivity, specificity, PPV, and NPV of 100% can distinguish SCC from SmCC. Similar to the study by Lyda, 34βE12 was positive in 8/20 of ADCs (40%). In all these eight specimens CK7 and TTF-1 were positive, too. The 34βE12 with 100% sensitivity, 60% specificity, 71.4% PPV, and 100% NPV distinguished ADC from SCC (P-value <0.0001). It is interesting to note that 34βE12 was mostly expressed in the areas of increased keratin formation, which was in contrast to P63 that was mostly expressed in the margins of squamous nests with poor differentiation. As a result, 34βE12 is recommended to distinguish SCC from ADC, and P63 is recommended to distinguish poorly-differentiated SCC from SmCC. 

CD56, also called neural cell adhesion molecule (NCAM), a SmCC indicator, was positive in 8/10 of SmCCs, 1/10 of ADCs, and 2/16 of SCCs in the study by Viberti(13). In other studies it was lowly or not expressed in NSmCCs(14, 15). In the current study, CD56 was positive in 18/20 of SmCCs and negative in ADCs and SCCs. Therefore, CD56 with 90% sensitivity, 100% specificity, 100% PPV, and 95.2% NPV can differentiate SmCC from NSmCCs.

In small biopsies, distinguishing SmCC from poorly-differentiated SCC is problematic and a panel of TTF-1, P63, HMWK (34βE12), and CD56 would be helpful. TTF-1, with a sensitivity of 87.5% and a specificity of 100%, can distinguish SmCC and ADC from SCC. CD56, with 100% sensitivity and PPV, confirmed the diagnosis of SmCCs, and negative 34βE12 ruled out the diagnosis of SCC (NPV 100%). P63, with 85% sensitivity, 97.5% specificity, 94.4% PPV, and 92.9% NPV, can distinguish SCC from SmCC and ADC.

TTF-1 and P63 with opposite immunoreactivity can differentiate SCC from ADC and SmCC and decrease false diagnosis rate due to aberrant immunoreactivity, and increase reliability. In addition, both of them are nuclear markers and in these specimens with little cytoplasm decrease false negativity. Moreover, they are more reliable than cytoplasmic markers (i.e. synaptophysin and chromogranin). Although typical ADC is not usually confused with SmCC and poorly differentiated SCC, it could be difficult to diagnose when the biopsy is small and the morphology shows small clusters or nests instead of glands. In such cases, CK7, with 100% sensitivity for ADC, and CD56, with 85% sensitivity for SmCC, can be added to the previous panel (TTF-1 and P63). On the other hand, 34βE12 is a very good marker with the sensitivity and specificity of 100% to differentiate SCC from SmCC, but not as good to differentiate it from ADC. Therefore, it is reasonable to use it with CK7, which has a sensitivity and specificity of 100% to differentiate ADC from SmCC. In a few cases in which the diagnosis of SmCC is probable, but TTF-1 is negative or P63 is positive, membranous staining of CD56 can be very helpful to diagnose SmCC (with 90% sensitivity). Moreover, tissue preservation factors such as areas with necrosis, and crush artifact can lead to decreased staining or false results and should be considered in interpretation of tumors without immunoreactivity.

The current study employed specimens with definite diagnosis and classification. The study aimed at evaluating the sensitivity and specificity of immunohistochemical markers employed to differentiate SCC, SmCC, and ADC.

The current study was approved by the Institutional Review Board *(IRB) *and Ethics Committee of Mashhad University of Medical Sciences (MUMS)*.*
